# Superimposition of virtual models using palatal rugae and maximum habitual intercuspation

**DOI:** 10.1590/2177-6709.29.2.e24spe2

**Published:** 2024-05-20

**Authors:** Isabella Simões HOLZ, Felipe A. R. CARVALHO, Rhita C. C. ALMEIDA

**Affiliations:** 1 Rio de Janeiro State University, Faculty of Dentistry, Department of Orthodontics (Rio de Janeiro/RJ, Brazil).

**Keywords:** Orthodontics, Treatment outcome, Dental models, Dental technology, Ortodontia, Efeitos do tratamento, Modelos dentais, Tecnologia dental

## Abstract

**Introduction:**

The superimposition of 3 dimensions (3D) digital models has been increasingly used for evaluating dental changes resulting from orthodontic treatment, and different superimposition techniques have been described. Although the maxilla has areas with greater stability for superimposition, such as the palatal rugae, there is still no reliable method for superimposing models of the lower arch.

**Objective:**

Therefore, this article aims to describe a technique for superimposing virtual models.

**Methods:**

To evaluate pre- and post-orthodontic treatment changes, the Geomagic Qualify 2013 software (3D Systems®, Rock Hill, South Carolina, USA) was used, with reference points in the maxilla, including the rugae and a reference area in the palate and midpalatal raphe. The lower arch was superimposed using the maximum habitual intercuspation (MHI) model as reference.

**Results and Conclusion:**

3D models superimposition using palatal rugae and MHI occlusion seems to offer satisfactory results in the interpretation of clinical changes at different follow-up moments in terms of development and/or orthodontic treatment.

## RESUMO

**Introdução:**

A sobreposição de modelos digitais em três dimensões (3D) tem sido cada vez mais utilizada como forma de avaliar as alterações dentárias decorrentes do tratamento instituído, e diferentes técnicas de sobreposição têm sido descritas. Apesar de a maxila apresentar áreas de maior estabilidade para sobreposição dos modelos, como as rugas palatinas, ainda não existe um método confiável para a sobreposição da arcada inferior.

**Objetivo:**

O presente artigo tem como objetivo descrever uma técnica de sobreposição de modelos virtuais.

**Métodos:**

Para avaliar as alterações pré e pós-tratamento ortodôntico, foi usado o *software* Geomagic Qualify 2013 (3D Systems®, Rock Hill, Carolina do Sul, EUA) , utilizando pontos de referência na maxila na região das rugas palatinas e uma área de referência na zona do palato e rafe palatina mediana. Já a arcada inferior foi sobreposta utilizando o modelo em máxima intercuspidação habitual (MIH) como referência.

**Resultados e Conclusão:**

A sobreposição de modelos utilizando as rugas palatinas e a oclusão em MIH parece oferecer resultados satisfatórios na interpretação das alterações clínicas entre momentos diferentes de acompanhamento, seja do crescimento/desenvolvimento e/ou resultados do tratamento ortodôntico.

## INTRODUCTION

The superimposition of three dimensions (3D) digital models has been increasingly implemented to assess the dental changes resulting from orthodontic treatment. Compared to the superimposition of two-dimensional (2D) cephalometric images and cone-beam computed tomography (CBCT), used over the past decades, the accuracy of measuring tooth positions and the absence of any radiation can be highlighted as the main benefits of the 3D method. Furthermore, the absence of tracing errors, the representation of the real size of the structures, and the absence of image distortion, mainly related to the dental surface and occlusal relationship, can also be added to the list of advantages.^1^ ^-^ ^3^

Different techniques of 3D models superimposition have been described in the literature, and are usually based on points^4^ ^-^ ^6^ and/or surface references.^2^ ^,^ ^4^ ^,^ ^7^ The palatal region of the maxilla is suggested as a stable parameter for superimposition due to its anatomy, and the region around the palatine rugae has considerable effect on a reliable superimposition result.^7^ In contrast, studies related to 3D models superimposition on the mandible are scarce and used mucogingival junction and alveolar bone as reference.^2^ ^,^ ^6^ ^,^ ^8^ ^,^ ^9^ However, alveolar bone remodeling is expected during growth and orthodontic treatment, especially when more complex movements are used. Thus, the current method has limitations and do not seems to provide reliable results.^2^ ^,^ ^10^

Therefore, a new digital model superimposition technique is necessary for an accurate evaluation of dental changes after orthodontic treatment, particularly in the lower arch. This article suggests a methodology for virtual models’ superimposition using two types of reference, based on landmarks and area and maximum habitual intercuspation (MHI) models as a parameter to minimize any discrepancy in the lower arch, since reference areas are scarce, compared to the maxilla. 

## DIGITAL MODEL

Dental digital models became a reality since its introduction in the late 20^th^ century through CAD/CAM (computer aided design/computer aided manufacture) system. The technology allows three-dimensional (3D) images acquisition and manipulation through a specific software, followed by 3D printing.^11^

The 3D models were initially obtained from scanned model, requiring traditional casting phase. Nowadays, with the intraoral scans’ popularization, dental arches are directed captured, minimizing anxiety and discomfort by the patient, with shorter chair-time and higher digital precision. In addition, there is no need for physical storage of dental models, allowing a more efficient clinical workflow, and improved patient acceptance.^12^ Clinical applications in orthodontics includes diagnosis, aligner production, customized devices, indirect bonding trays and treatment results.^13^

Tooth movement performance through orthodontic treatment can be assessed three-dimensionally by 3D model superimposition, and provide more reliable and reproducible outcomes, compared to linear measurements on dental casts, such as overjet, overbite, arch width,^14^ or other images exams. However, to improve the reliability of the superimposition, a stable reference in both arches must be defined. 

## MAXILLARY MODEL SUPERIMPOSITION

For any superimposition, a stable anatomic structure is required as reference, and the palatal anatomy seems to provide reliable results in the maxilla. However, although the literature is vast in works that used reference points and regions in the maxilla, they differ regarding which region presents more stability, and none had been designated as gold standard.^15^

Different palatal regions have been suggested as parameters for superimposition, and the reference area used has considerable effect on the superimposition result, as well as on the number of reference landmarks.^7^ Some studies suggested concomitant palatal rugae alterations in the anterior and lateral end area following tooth movement, especially in patients who underwent tooth extraction.^15^ ^,^ ^16^

The superimposition of the upper arch is achieved with the demarcation of reference points based on reliable regions reported in previous studies, such as the palatine rugae and incisive papilla, and reference surface in the maxilla rugae region and midpalatal raphe. The areas in the maxilla that seems to be the most stable for superimposition are the medial thirds of the third palatine rugae, including a 5 mm area dorsal to it,^16^ and a region that includes the entire rugae, with lateral margins located within 5 mm of the gingival margins, and a distal margin that does not extend beyond the first molars.^4^ ^,^ ^10^ ^,^ ^15^ ^,^ ^17^

## MANDIBULAR MODELS SUPERIMPOSITION

Opposite to the upper arch, the mandible is scarce in stable structures. Studies related to 3D models superimposition on the mandible^2^ ^,^ ^6^ ^,^ ^8^ ^,^ ^9^ reported different parameters for the mandibular arch superimposition^2^ ^,^ ^8^ ^,^ ^9^, including the mucogingival junction^8^ ^,^ ^9^ and mandibular torus as references.^2^

An et al.^2^ assessed the reliability of the lingual and buccal mandibular surfaces as reference areas for digital model superimposition in patients with premolar extraction with and without torus. The authors concluded that the alveolar bone either of anterior or posterior teeth is inappropriate as reference for mandibular superimposition models in patients without mandibular torus. Also, in premolar extraction cases, the lower anterior teeth seem to suffer more remodeling than the posterior teeth.

Contrarily, Ioshida et al.^9^ used landmarks at the mucogingival junction to assess pre- and post-treatment changes in patients with lower anterior crowding, and considered the method reliable and reproducible, compared to CBCT. However, patients who are candidates for orthodontic treatment have different malocclusions complexity, including patients with extractions. 

The use of the alveolar bone as a reference for superimposition is not adequately supported by studies up to date, since an alveolar bone remodeling is expected during growth and orthodontic treatment, especially when more complex movements are used. Thus, the described approaches have limitations and do not seem to provide reliable results.^2^ ^,^ ^10^

## SOFTWARES FOR DIGITAL MODEL SUPERIMPOSITION

Different methods of 3D models superimposition have been described in the literature, and are usually based on points^4^ ^-^ ^6^ and/or on surface references,^2^ ^,^ ^4^ ^,^ ^7^ defined by the operator associated with computer-based algorithm. The landmark-based superimposition requires manual identification of several corresponding anatomical landmarks on both models for registering; whereas in the surface-based ones, the approach usually requires the selection of a reference area.^7^ These parameters are used for an initial approximation of the models, complemented by the global registration provided by the software. The combination of reliable reference points and areas, together with the software’s iterative closest points (ICP) algorithm, seems to provide good reliability for the method. 

Slicer CFM,^6^ ^,^ ^9^ ^,^ ^18^ OMSA,^4^ Final Surface,^17^ OrthoAnalizer, Geomagic^19^ ^-^ ^21^ and Compare^8^ are examples of softwares for digital model superimposition described in the available literature. They differ regarding costs, the methods for measuring 3D movements, time and complexity of the superimposition process.^8^

Some studies used the Geomagic as the software of choice for models’ superimposition.^8^ ^,^ ^19^ ^-^ ^21^ According to its developer, the orientation for superimposition is the use of reference points followed by the “best fit” mode for global registration. Adel et al.^8^ used the recommended methodology contemplating three reference points in the medial portion of the palatal rugae in the upper arch, and three points in the mucogingival junction between the first and second premolars, second premolars and first molars, and between first and second molars. The methodology showed excellent agreement for the measurements of the upper arch and good agreement for the lower arch. However, despite the acceptable agreement, the reference points used in the lower arch may not be stable and have limitations, particularly in cases in which movement of the lower teeth is large.

## CLINICAL SITUATIONS FOR MODEL SUPERIMPOSITION

**Figure 1:**
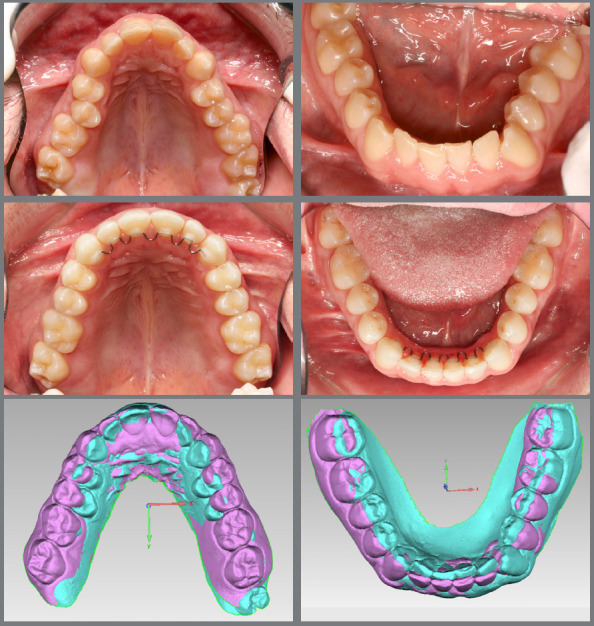
Pretreatment (blue) and post-treatment (purple) 3D models superimposition and photographs. The treatment plan included arch expansion and teeth decompensation for orthognathic surgery.

**Figure 2:**
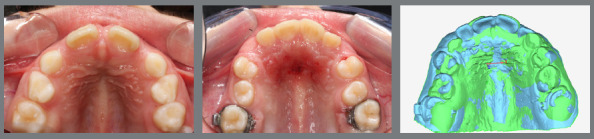
Pretreatment (green) and post-treatment (blue) 3D models superimposition and photographs after arch expansion and molar distalization with intraoral distalizer, to create space for the upper right second premolar.

**Figure 3:**
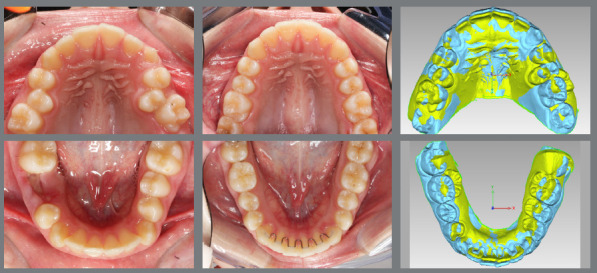
Pretreatment (green) and post-treatment (blue) 3D models superimposition and photographs. The treatment comprised upper arch contraction and lower arch expansion, to correct a Brodie crossbite.

**Figure 4:**
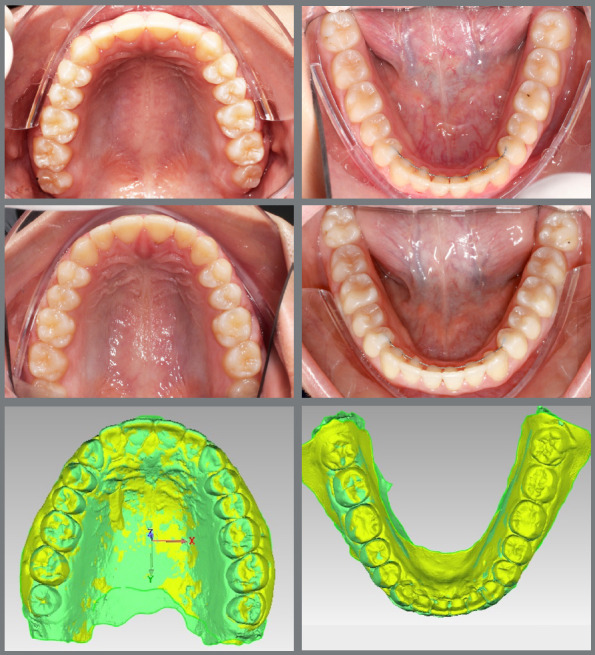
Photographs and 3D models superimposition to evaluate treatment stability after orthodontic treatment.

The main objective of digital model superimposition is to assess treatment outcomes - such as teeth alignment, arch expansion (Fig 1), distalization (Fig 2), arch contraction (Fig 3) -, to evaluate capabilities and limitations of appliances and mechanics. Moreover, model superimposition following orthodontic treatment is useful to evaluate treatment stability (Fig 4).

## SUPERIMPOSITION SEQUENCE

**Figure 5:**
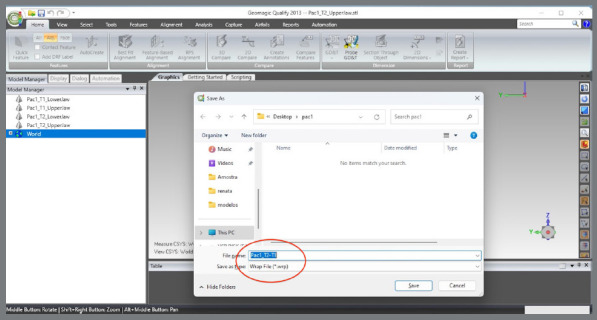
Import the models into the software and save in the .WRP format.

**Figure 6:**
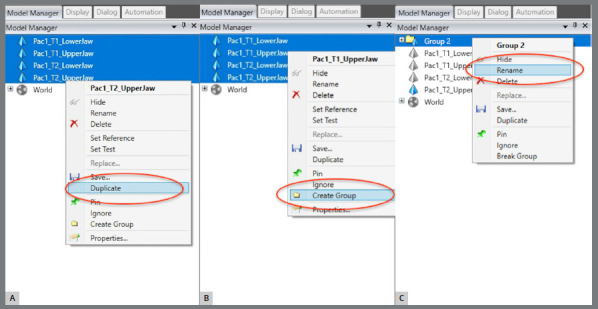
A) Models selection and duplication. B) The group was created; C) and renamed as “Originals”.

**Figure 7:**
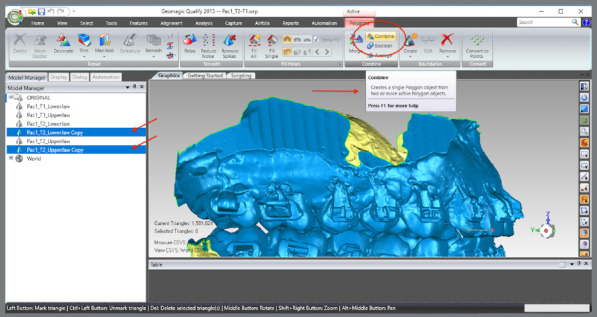
Duplication of T2 models and combination of copies to create a file on MHI occlusion.

**Figure 8:**
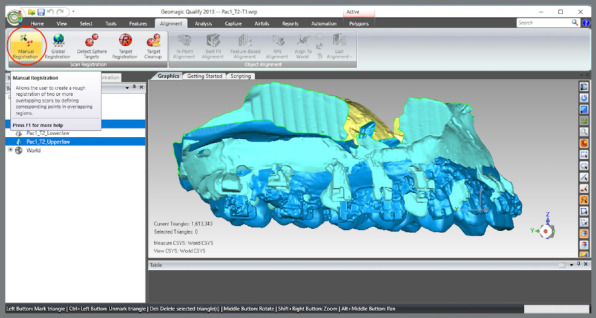
Selection of T1 and T2 models of the upper arch and selection of the item for manual registration.

**Figure 9:**
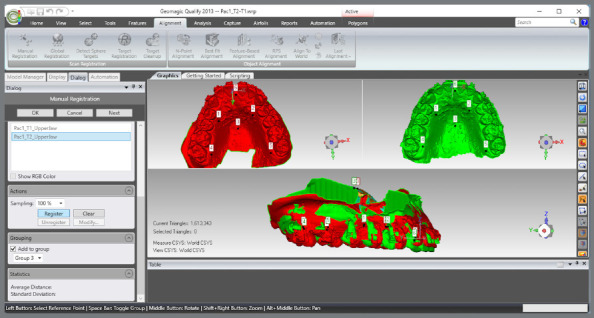
Landmarks definition for superimposition of T2 with T1 for the upper arch.

**Figure 10:**
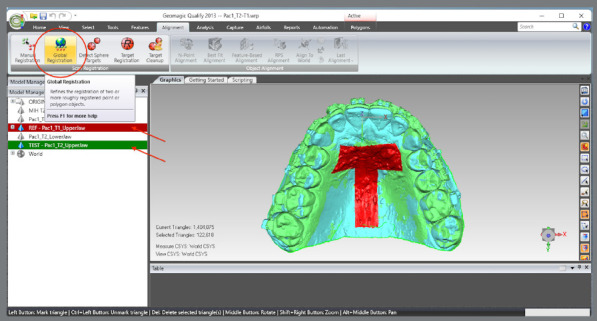
Demarcation of the area of interest for superimposition, according to the global registration.

The scanned models were imported in STL format into the Geomagic Qualify 2013 software (3D Systems^®^, Rock Hill, South Carolina, USA), aiming to standardize the areas to be evaluated between the pre- (T1) and post-treatment (T2) models. The following sequence was implemented:

1. The upper and lower models for the pre- (T1) and post-treatment (T2) times were imported.
2. A single WRP file (Wrap file) comprising the four STL files was created (Fig 5).
3. The four models were imported, and an “Original” group (for backup) was created (Fig 6).
4. A MHI model copy of T2 was created as a parameter for lower arch superimposition:
  a) Duplication of T2 models and combination of copies to create a file on MHI occlusion (Fig 7).
5. The upper models were aligned by landmark points:
  a) T1 and T2 models were selected, and the manual registration proceeded (Fig 8).
  b) The mode “n-point Registration” was selected.
  c) T1 model was defined as “fixed” (as a parameter for the superimposition) and T2, as “floating” (it will be approximated to T1) (Fig 9).
  d) Six points were marked in both models: two in the distal region of the second maxilla rugae; one at the incisive papilla region; one at the middle third of the region of the maxilla raphe; and two points in the cervical-lingual region of the second molars, and manual registration was proceeded (Fig 9).
6. The upper models were aligned by marking the reference area (Fig 10):
  a) The upper models were selected.
  b) A “T” shaped reference area including the palatal rugae and the midpalatal raphe was selected. The selection mode must be “Selection Mode” > “Select Through,” and the “Selection Tool” must be “Polyline.
  c) The global alignment was done.
7. The T2 MHI model was aligned with the T2 maxillary model, previously registered at T1:
  a) T2 model was selected and defined as “reference”. The area of ​​the model covering the dental and gingival surfaces, except for the occlusal surfaces, was selected.
  b) MIH model was selected and defined as “test”. The area of ​​the model covering the dental and gingival surfaces, except for the occlusal surfaces, was selected.
  c) The MIH model was selected and proceeded with: Alignment > Best fit Analysis.
  d) Now, both models were selected, and the selections made previously were cleared. The area of ​​the model covering the dental and gingival surfaces, except for the occlusal surfaces, were re-select and best fit analysis proceeded, but at this time, the “Check Symmetry”, “Fine Adjustments Only” and “High Precision Fitting” options were selected.
8. The same process was repeated for the lower arch, but, at this point, the MHI model was set as “reference,” and the lower T2, as “test.”
9. Excesses of the upper and lower models were cut:
  a) The two upper or lower models were selected.
  b) The area to be cropped and deleted was selected (Note that in this step the selection mode must be “Selection Mode” > “Select Through,” and the “Selection Tool” must be “Polyline”) (Fig 12A).
  c) The selected part was deleted (Fig 12B).
10. The coordinate system was generated:
  a) The axial plane was defined by selecting three points in the upper model, on the mesiolingual cusps of teeth #16 and #26, and between teeth #11 and #21, at T1 (Fig 13).
  b) The centroid point was defined from the selection of the upper model (in this step, the entire upper model must be selected, after which the sequence “Features” > “Point” > “Centroid” must be chosen) (Fig 14).
  c) The sagittal plane was defined by selecting three points in the midpalatal raphe (anterior, middle, and posterior thirds). In the point 2, the centroid was used as a reference (Fig 15).
  d) The global models’ reference was defined according to the coordinate plane:
    1. The YZ plane was defined as the sagittal plane (Fig 16A).
    2. The XY plane was defined as the axial plane (Fig 16B).
    3. The created coordinate system was added to “World” (Fig 17).
    4. In “World”, the “World CSYS” was “hidden” and the created coordinate system was defined as active CYS (Fig 17).
11. The differences between registered models were quantified as following:
  a) The points for measuring differences in position between T1 and T2 were marked according to the areas of interest.
  b) The items “Features” > “Point” > “Parameters” were selected to mark the points in the area of ​​interest (Fig 18A).
  c) From each point marked, the position in the coordinate system in the directions x (lateral), y (anteroposterior), z (superoinferior) was recorded (Fig 18B and 18C).
  d) The difference in position between the times was obtained from the difference between the values ​​of the position of the points of T2-T1 in the directions x (lateral), y (anteroposterior), z (superoinferior), to obtain the displacement magnitude of certain regions of these structures, as well as the direction in which these movements occurred (Fig 18D).

Another way to perform distance analysis is through the function “Analysis” > “Distance” > “Measure distance”, and select the two points to be measured. To do this, the T1 and T2 models must be selected, duplicated and combined. 

[Fig f11]

**Figure 11: f11:**
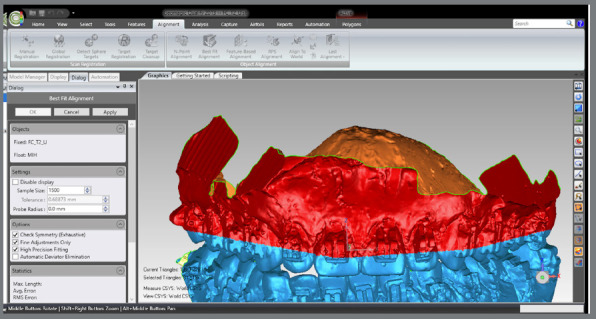
Demarcation of the area of interest for the overlay, according to the “best fit”.

**Figure 12:**
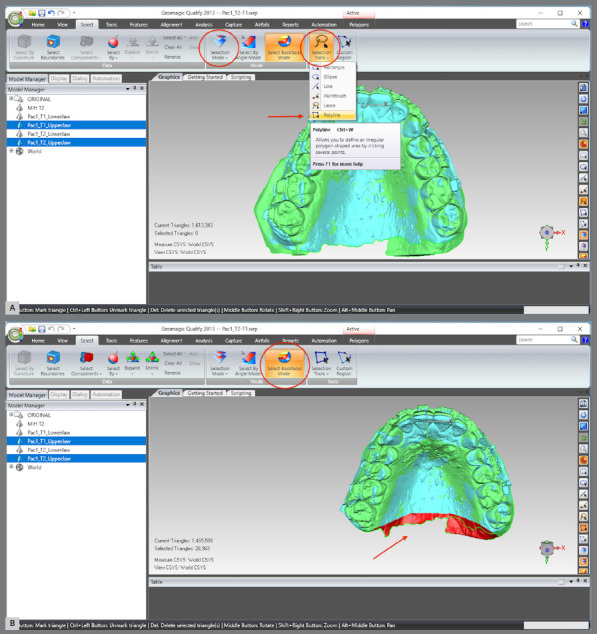
A) Selection of both upper models and B) demarcation and trimming the excesses for both models.

**Figure 13:**
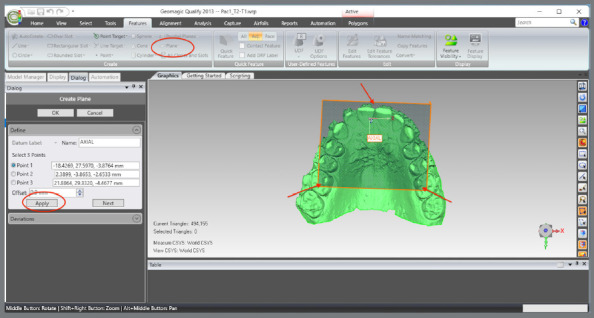
Definition of the axial plane from the demarcation of three points, two on the mesiolingual cusps of teeth #16 and #26, and one between the central incisors.

**Figure 14:**
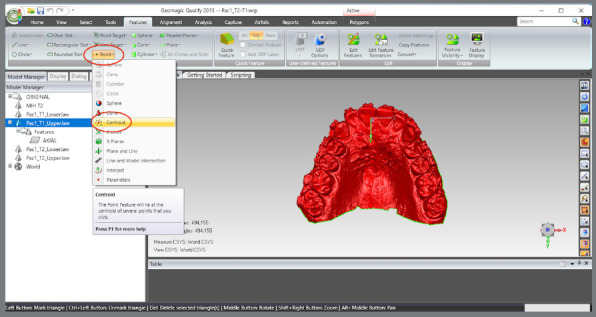
Centroid point definition by the model selection.

**Figure 15:**
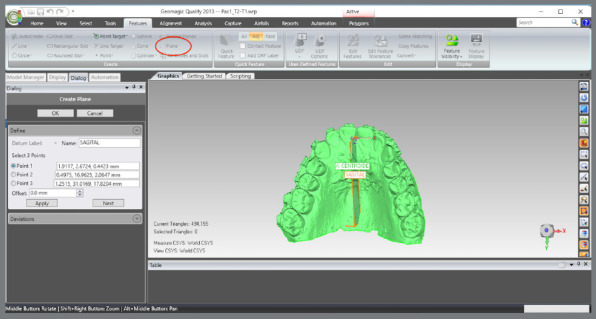
Sagittal plane definition by marking a point close to the incisive papilla, another at the centroid position, and a third at the distal end of the midpalatal raphe.

**Figure 16:**
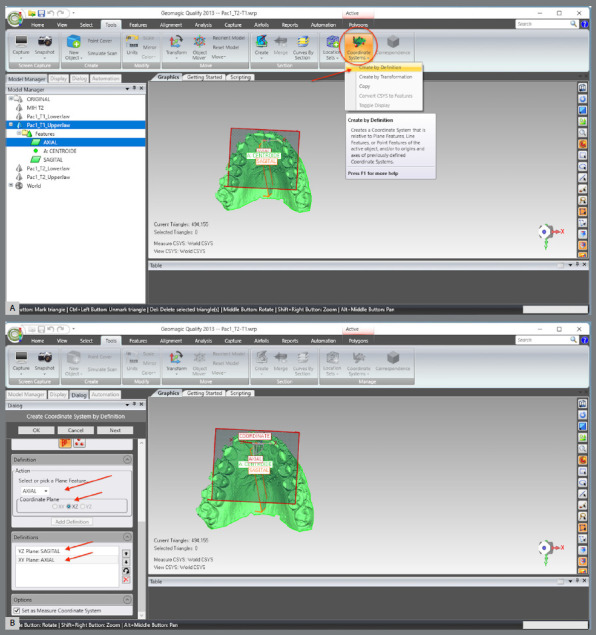
Definition of the coordinate system and definition of the axial and sagittal planes.

**Figure 17:**
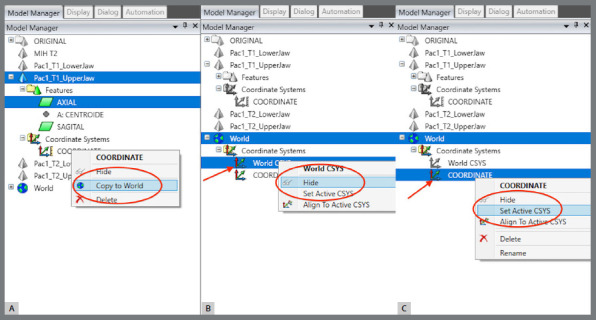
Definition of the coordinate system: A) select and copy the coordinate system created for “World”; B) in “World”, “Hide” the “World CSYS” and C) define the created coordinate system as active.

**Figure 18:**
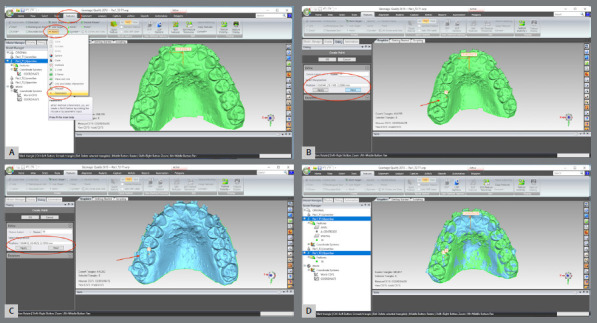
A) Function selection for marking points: “Features” > “Point” > “Parameters”; marking of the points in the chosen region for measuring the displacement in tooth #16 in T1 (B) and T2 (C). D) Superimposition of the models for the evaluation of tooth displacement through the marked points.

**Figure 19:**
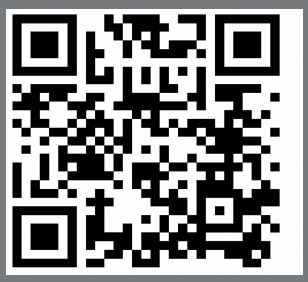
Scan the QR code to watch the superimposition sequence video.

For more details about the method, access the video using the “QR code” at the Figure 19.

## LIMITATIONS OF THE METHOD

The limitations in the described approach are mainly relevant for the lower arch, and are inherent to the absence of stable structures for mandibular models’ comparison. Therefore, when interpreting these changes, the effects of growth and consequent repositioning of mandible space cannot be ignored, particularly in the vertical direction. In fact, this same problem would be analogous to what occurs with superimposition of lateral cephalograms and CBCT where the skull base is used as a static parameter.^22^ Furthermore, a study to validate the methodology is necessary for the proper scientific applicability of measuring pre- and post-treatment changes using the proposed method. 

## CONCLUSIONS

The superimposition of models using palatal rugae and occlusion in MHI seems to offer satisfactory results in the interpretation of clinical alterations between different follow-up moments, whether in terms of development and/or of orthodontic treatment. Studies to validate the reproducibility of the methodology are necessary for the applicability of digital models’ superimposition. 
